# Validity of self-reported weight, height, and BMI in mothers of the research Birth in Brazil

**DOI:** 10.11606/S1518-8787.2017051006775

**Published:** 2017-11-16

**Authors:** Roberta Gabriela Pimenta da Silva Araújo, Silvana Granado Nogueira da Gama, Denise Cavalcante de Barros, Cláudia Saunders, Inês Echenique Mattos

**Affiliations:** I Fundação Oswaldo Cruz. Escola Nacional de Saúde Pública Sergio Arouca. Departamento de Epidemiologia e Métodos Quantitativos em Saúde. Rio de Janeiro, RJ, Brasil; II Fundação Oswaldo Cruz. Escola Nacional de Saúde Pública Sergio Arouca. Centro de Saúde Escola Germano Sinval Faria. Rio de Janeiro, RJ, Brasil; III Universidade Federal do Rio de Janeiro. Instituto de Nutrição Josué de Castro. Rio de Janeiro, RJ, Brasil; IV Fundação Oswaldo Cruz. Escola Nacional de Saúde Pública Sergio Arouca. Programa de Pós-Graduação em Epidemiologia em Saúde Pública. Rio de Janeiro, RJ, Brasil

**Keywords:** Pregnant Women, Body Weight, Body Height, Body Mass Index, Self-Assessment, Reproducibility of Results, Validation Studies, Gestantes, Peso Corporal, Estatura, Índice de Massa Corporal, Autoavaliação, Reprodutibilidade dos Testes, Estudos de Validação

## Abstract

**OBJECTIVE:**

To evaluate the accuracy of information on pre-gestational weight, height, pre-gestational body mass index, and weight at the last prenatal appointment, according to maternal characteristics and sociodemographic and prenatal variables.

**METHODS:**

The study was developed using data from the face-to-face questionnaire and prenatal card (gold standard) of the study “Birth in Brazil, 2011–2012”. To evaluate the differences between the measured and self-reported anthropometric variables, we used the the Kruskal-Wallis test for the variables divided into quartiles. For the continuous variables, we used the Wilcoxon test, Bland-Altman plot, and average difference between the information measured and reported by the women. We estimated sensitivity and the intraclass correlation coefficient.

**RESULTS:**

In the study, 17,093 women had the prenatal card. There was an underestimation of pre-gestational weight of 1.51 kg (SD = 3.44) and body mass index of 0.79 kg/m^2^ (SD = 1.72) and overestimation of height of 0.75 cm (SD = 3.03) and weight at the last appointment of 0.22 kg (SD = 2.09). The intraclass correlation coefficients (ICC) obtained for the anthropometric variables were: height (ICC = 0.89), pre-gestational weight (ICC = 0.96), pre-gestational body mass index (ICC = 0.92), and weight at the last appointment (ICC = 0.98).

**CONCLUSIONS:**

The results suggest that the mentioned anthropometric variables were valid for the study population, and they may be used in studies of populations with similar characteristics.

## RESUMO

**OBJETIVO:**

Avaliar a acurácia das informações de peso pré-gestacional, estatura, índice de massa corporal pré-gestacional e peso na última consulta de pré-natal, segundo características maternas, variáveis sociodemográficas e de pré-natal.

**MÉTODOS:**

O estudo foi desenvolvido com dados do questionário face a face e do cartão da gestante (padrão-ouro) do estudo “Nascer no Brasil, 2011–2012”. Para avaliar as diferenças entre as variáveis antropométricas medidas e referidas, utilizou-se o teste de Kruskal-Wallis para as variáveis dividas em quartis. Para as variáveis contínuas, adotou-se o teste de Wilcoxon, gráficos de Bland e Altman, diferença média entre as informações medidas e referidas pelas mulheres. Estimou-se a sensibilidade e o coeficiente de correlação intraclasse.

**RESULTADOS:**

No estudo, 17.093 mulheres possuíam cartão de gestante. Observou-se uma subestimação do peso pré-gestacional em 1,51 kg (DP = 3,44) e do índice de massa corporal em 0,79 kg/m^2^ (DP = 1,72), e superestimação da estatura em 0,75 cm (DP = 3,03) e do peso na última consulta em 0,22 kg (DP = 2,09). Os coeficientes de correlação intraclasse (CCIC) obtidos para as variáveis antropométricas foram: estatura (CCIC = 0,89), peso pré-gestacional (CCIC = 0,96), índice de massa corporal pré-gestacional (CCIC = 0,92) e peso na última consulta (CCIC = 0,98).

**CONCLUSÕES:**

Os resultados sugerem que as variáveis antropométricas referidas foram válidas para a população de estudo, e podem ser utilizadas em estudos com populações que tenham características semelhantes.

## INTRODUCTION

The evaluation of the anthropometric nutritional status is part of the clinical practice and is frequently used in health research. Weight and height are important instruments for the anthropometric evaluation of the population, since they are good predictors of the functional conditions of the organism, morbidity, and mortality^25^. During gestation, these measures are useful anthropometric indicators for the prevention of unfavorable maternal outcomes, such as inadequate weight gain, gestational diabetes, and hypertension, as well as problems with the child, such as macrosomia and perinatal death^8^ ^,^ ^11^ ^,^ ^25^.

Pre-gestational body mass index (BMI) is one of the most relevant indicator to monitor the nutritional status of women during pregnancy. The Institute of Medicine (IOM) and the Ministry of Health of Brazil recommend the classification of BMI to estimate the appropriate total gestational weight gain for each woman, which may reduce the number of complications for the mother-child binomial^6^ ^,^ ^11^.

In addition, the IOM recommends that validation studies should be developed for weight, height, and BMI at different stages of the gestational period to support guidelines proposed for weight gain during pregnancy^11^.

These measurements are obtained using easy to medium complexity techniques that are non-invasive, and the direct measurement is the preferred way to obtain these data. However, because of problems such as lack of equipment and high cost of research, population-based epidemiological studies have used reported measures of weight and height as a valid alternative to those acquired directly, since they produce proxy results of real values^9^ ^,^ ^11^ ^,^ ^18^.

Given the importance of these measures, by verifying the validity of this information we can help in the correct classification of the nutritional status of women, allowing the use of reported data for a population sample with the same characteristics.

This study aimed to evaluate the accuracy of pre-gestational weight, height, pre-gestational BMI, and weight at the last prenatal appointment reported by women, according to maternal characteristics and sociodemographic and prenatal variables.

## METHODS

This is a descriptive study of the validity of the anthropometric information of the research “Birth in Brazil, 2011–2012”, a research of national scope and hospital basis carried out with mothers and their babies, between February 2011 and October 2012, in Brazil. The sample was selected in three stages. The first one consists of hospitals with 500 or more births a year, stratified by the five macro-regions of the country, location (capital or non-capital), and type of hospital (private, public, and mixed). The second stage consists of the number of days in each hospital (minimum of seven days), and the third one consists of the mothers. In each of the 266 hospitals sampled, 90 mothers were interviewed, amounting to 23,894 individuals. More information about the sample design is detailed in Vasconcellos et al.^24^

Face-to-face interviews were carried out with the mothers during hospitalization, data were extracted from the woman’s and the newborn’s medical records, and pregnancy prenatal care cards were photographed^7^ ^,^ ^15^.

In order to meet the objective of this validation study, we considered as eligible women who had the prenatal card, from which we obtained the reference values (gold standard) for the variables: pre-gestational weight in kilograms (kg), height in centimeters (cm), weight at the last appointment (kg), and pre-gestational BMI obtained using the formula [pre-gestational weight (kg) / height^2^ (m^2^)].

Another inclusion criterion was the mother who answered at least one of the questions in the face-to-face questionnaire on biometric information, corresponding to the reported measures “What was your weight before pregnancy?”, “What was your weight at the last prenatal appointment?”, “What is your height?”.

The variables of interest for the validation study were: pre-gestational weight, height, weight at the last prenatal appointment, pre-gestational BMI, hospital macro-region, type of service in which the prenatal appointments were performed (public, private, or both), age group (< 20, 20–34, ≥ 35 years), self-reported race (black, brown, white, yellow, and indigenous), marital status (living with or without partner), education level (incomplete basic education, complete basic education, complete high school, complete higher education), economic classification (class A or B, class C, class D or E)^1^, number of prenatal appointments (1–3, 4–5, 6 or more), and number of previous pregnancies (none, one, two, three or more).

**Figure 1:**
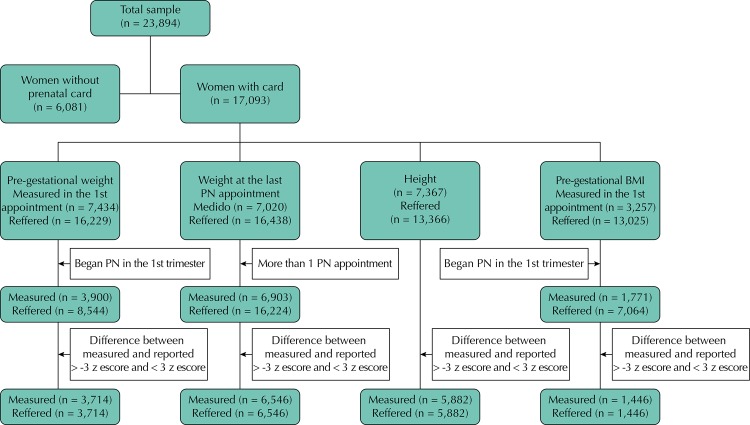
Flowchart to obtain the sample.

PN: prenatal; BMI: body mass index

Figure 1 represents the flowchart with the inclusion criteria used to obtain the final sample. To exclude the outliers from the measured and reported anthropometric variables, we chose to use the parameters proposed by Larsen et al.^13^, and we included the classifications in the interval established by ± 3 z-score of the difference between the measured and reported variables in the analysis and results presented. Therefore, the influence of these points on the agreement of the information was evaluated using the values presented by weighted kappa, using the quadratic weight, which is close to the intraclass correlation coefficient (ICC)^12^.

We assessed the validity of the analysis and the potential for selection bias given 25% that not be include, because they don’t have prenatal card. We compared the characteristics of the sample of cases not include with the ones included. For this step of the analyzes, we considered the complex sampling project, applying sample weights and corrections to give consistency between population estimates^24^.

In the research Birth in Brazil, the probability of selecting women was different; therefore, we needed to create sample weights so that prevalence results could be representative. For the concordance analyses, we used the original sample data, as the purpose of this study was to validate the answers and not to evaluate some type of prevalence. Therefore, we did not weight them in this step. The chi-square test was used for the concordance analyses.

We calculated the average differences of the anthropometric variables by subtracting the values of the reported variables from the values of the measured variables. Therefore, a negative value indicates overestimation of the reported variable in relation to the measured one and a positive value indicates underestimation^9^ ^,^ ^21^.

The values of the anthropometric variables were tested using the Kolmogorov-Smirnov test to verify normality. We used the Kruskal-Wallis test to evaluate the average difference of the reported variables (pre-gestational weight, height, weight at the last appointment, and BMI) in relation to the measured variables (reference), divided into quartiles. Wilcoxon signed-rank test was used to identify the differences between the averages of the direct and reported information of the analyzed variables, in their continuous distribution. We chose to use non-parametric tests, since the variables of interest did not have a normal distribution.

For the validation of the measurements, we estimated the sensitivity of the anthropometric variables in relation to pre-gestational weight, weight at the last appointment, height, and pre-gestational BMI, divided into quartiles (P25 – 1st quartile, P25-50 – 2nd quartile, P50-75 – 3rd quartile, and P75 – 4th quartile), and the variations in sensitivity were evaluated according to the maternal, prenatal, socioeconomic, and demographic variables. Measured and reported pre-gestational BMI were categorized according to what is proposed by the World Health Organization^25^: low-weight < 18.5 kg/m^2^, normal range 18.5–24.9 kg/m^2^, overweight 25.0–29.9 kg/m^2^, and obesity > 30 kg/m^2^.

We used the intraclass correlation coefficient (ICC), which takes into account systematic bias, two-way mixed, with absolute concordance for the continuous anthropometric variables. We evaluated the existence of interobserver reproducibility, that is, if the tests obtained the same result with different methods, for comparability purposes. To evaluate the observed values, we used the criteria of Landis and Koch^12^, in which ICC < 0 is poor; from 0 to 0.20, weak; from 0.21 to 0.40, probable; from 0.41 to 0.60, moderate; from 0.61 to 0.80, substantial; and from 0.81 to 1.00, almost perfect. We also used the Bland-Altman plot^2^ to evaluate the possible systematic patterns and errors of the average differences between the measured and reported variables (ordinate axis), in relation to their average (abscissa axis).

We used Pearson’s chi-square test to analyze the distribution between those with or without accurate pre-gestational weight, weight at the last appointment, height, and pre-gestational BMI. Accuracy was considered acceptable when the average difference between the measured and reported values for weight if within ± 2 kg, for height if within ± 2 cmand between ± 1.4 kg/m^2^ for BMI_5_. The statistical level of significance adopted was 5%.

Statistical analysis was performed using the software IBM SPSS for Windows 8, version 20, and winpepi, version 11.43.

The study has been approved by the Ethics Committee of the Escola Nacional de Saúde Pública (92/10) under CAE 0096.0.031.000-10.

## RESULTS

A total of 17,093 (71.5%) women had the prenatal card, approximately 23% had pre-gestational weight measured in the first trimester, measured height was present in 43% of the cards, allowing the calculation of the measured pre-gestational BMI in 19.1% of them, and 41.1% of the cards had weight at the last prenatal appointment (Figure 1). For the validation study, of the percentages presented previously, we considered the anthropometric variables within the range of ± 3 z-score, from which we found the record of 50% for measured pre-gestational weight, 79.8%, for height, 44% for pre-gestational BMI, and 93% for weight at the last appointment.

Of those who went to prenatal appointments, 77% in the SUS and 69.5% in the private sector had the prenatal card at the time of the interview. Women of the South and Southeast regions, adolescents, those from class C+D or E, brown, and primigravida were more likely to have the card (data not shown).

The women who reported their height tended to overestimate it by an average of 0.75 cm when compared to the measurements. We verified that the weight reported at the last appointment is close to the one measured, with a difference of 0.2 kg (Table 1).

**Table 1:** Average values of height, pre-gestational weight, weight at the last appointment, and pre-gestational BMI, according to SD and quartiles. Brazil, 2011–2012.

Variable		n	SD	Quartile				Total

				1st	2nd	3rd	4th	
Height	Average measured	5,882	6.774	149.8	155.6	160.4	167.7	158.57
	Average reported	5,882	6.993	150.0	156.5	162.5	169.9	159.32
	Average height difference^c^	5,882	3.033	-4.7^a^	-1.0^a^	0	2.9^a^	-0.750^b^
Pre-gestational weight	Average measured	3,714	12.822	48.0	56.0	63.9	80.1	61.98
	Average reported	3,714	12.654	46.6	54.3	61.6	77.9	60.47
	Average pre-gestational weight difference^c^	3,714	3.436	-3.0^a^	0	1.8^a^	6.6^a^	1.510^b^
Weight at the last appointment	Average measured	6,546	13.396	57.2	65.9	74.6	91.4	72.436
	Average reported	6,546	13.282	57.5	66.6	75.2	91.1	72.654
	Average weight difference at the last appointment^c^	6,546	2.092	-2.6^a^	-0.6^a^	0	2.4^a^	-0.218^b^
Pre-gestational BMI	Average measured	1,446	4.835	19.4	22.1	25.3	31.2	24.479
	Average reported	1,446	4.717	18.7	21.6	24.2	30.2	23.69
	Average pre-gestational BMI difference^c^	1,446	1.723	-1.3^a^	0	0.8^a^	3.1^a^	0.790^b^

n: total of mothers per variable; BMI: body mass index; SD: standard deviation

^a^ Significant differences, according to Kruskal-Wallis test with p < 0.05.

^b^ Significant differences, according to Wilcoxon test, for continuous variables, with p < 0.05.

^c^ Average difference: difference between the measured and reported variables, calculated for each woman within the quartiles, reported as average values in the table. Therefore, there was underestimation if the value is positive and overestimation if the value is negative.

The mothers in 1.51 kg and 0.80 kg/m^2^, respectively, underestimate pre-gestational weight and pre-gestational BMI. From the second quartile, we can notice a difference in the average of the reported values.

The differences between the measured and reported variables are greater in the extremes, the first and fourth quartiles (Q). We highlight the accuracy of the anthropometric variables; the highest, 76%, was found for weight at the last prenatal appointment, and the lowest, 50%, for pre-gestational weight (Table 2).

**Table 2:** Distribution of mothers by variables selected for accuracy according to the variables of pre-gestational weight, height, weight at the last appointment, and pre-gestational BMI. Brazil, 2011–2012.

Variable	Height			p	Pre-gestational weight			p	Weight at the last appointment			p	BMI			p
			
	Accuracy*		Total		Accuracy*		Total		Accuracy*		Total		Accuracy*		Total	
			
	N	% per category	n		N	% per category	N		N	% per category	N		N	% per category	N	
Place of the PN				< 0.05				< 0.05				0.158				0.091
Public service	3,388	63.9	5,303		1,305	47.1	2,772		3,928	75.9	5,176		794	62.1	1,279	
Private service	223	70.8	315		467	59.3	788		894	78.6	1,138		66	69.5	95	
Both	158	64.0	247		75	50.7	148		172	76.4	225		52	72.2	72	
Total	3,769	64.3	5,865		1,847	49.8	3,708		4,994	76.4	6,539		909	62.8	1,446	
Geographic region				< 0.05				< 0.05				< 0.05				0.129
North	505	63.8	792		113	42.5	266		359	67.6	531		114	68.7	166	
Northeast	1,209	63.5	1,903		456	44.7	1,019		1,531	76.6	2,000		279	59.4	470	
Southeast	1,250	62.4	2,002		878	54.4	1,615		2,067	77.8	2,658		298	63.5	469	
South	568	70.0	811		336	49.6	677		861	77.8	1,107		164	62.8	261	
Midwest	248	66.0	376		65	47.8	136		182	72.5	251		56	70.9	79	
Total	3,780	64.2	5,884		1,848	49.8	3,713		4,987	76.4	6,547		908	63.0	1,445	
Age group (years)				0.283				0.272				< 0.05				0.347
12–19	865	66.0	1,310		281	46.8	600		1,039	72.5	1,434		172	62.1	277	
20–34	2,638	63.8	4,132		1,383	50.3	2,751		3,496	77.4	4,515		657	62.6	1,049	
> 34	273	62.8	435		185	51.1	362		459	77.5	592		83	69.2	120	
Total	3,776	64.3	5,877		1,849	49.8	3,713		4,994	76.3	6,541		912	63.1	1,446	
Race				< 0.05				< 0.05				0.276				< 0.05
White	1,131	66.8	1,692		746	54.1	1,380		1,721	77.9	2,208		331	70.9	467	
Black	301	61.2	492		110	42.5	259		424	76.4	555		46	44.2	104	
Brown	2,277	63.2	3,602		975	48.0	2,032		2,789	75.5	3,693		526	61.2	859	
Yellow	53	76.8	69		16	48.5	33		47	72.3	65		8	57.1	14	
Indigenous	17	65.4	26		2	20.0	10		17	77.3	22		1	33.3	3	
Total	3,779	64.3	5,881		1,849	49.8	3,714		4,998	76.4	6,543		912	63.0	1,447	
Marital status of the mother				0.946				0.056				0.067				0.255
Without partner	724	64.4	1,125		292	53.6	545		914	74.4	1,229		121	66.9	181	
With partner	3,055	64.2	4,755		1,557	49.1	3,168		4,083	76.8	5,314		791	62.5	1,266	
Total	3,779	64.3	5,880		1,849	49.8	3,713		4,997	76.4	6,543		912	63.0	1,447	
Education of the mother				< 0.05				< 0.05				< 0.05				< 0.05
Incomplete BE	1,008	62.8	1,604		319	40.1	795		1,390	74.3	1,872		183	52.6	348	
Complete BE	1,175	63.2	1,858		440	46.2	953		1,368	75.3	1,817		278	63.9	435	
Complete HS	1,421	65.2	2,178		891	54.0	1,649		1,893	77.9	2,431		394	66.7	591	
Complete HE and more	164	74.2	221		191	63.7	300		324	81.8	396		54	81.8	68	
Total	3,768	64.3	5,861		1,841	49.8	3,697		4,975	76.4	6,516		909	63.0	1,442	
Economic class				< 0.05				< 0.05				0.189				0.073
Classes A+B	581	69.2	839		507	55.8	909		984	78.3	1,257		162	69.5	233	
Class C	2,106	63.4	3,321		968	48.4	1,999		2,683	75.9	3,534		534	62.0	861	
Classes D+E	1,069	63.9	1,673		366	46.6	786		1,291	75.7	1,705		209	60.9	343	
Total	3,756	64.4	5,833		1,841	49.8	3,694		4,958	76.3	6,496	0.178	905	63.0	1,437	
Number of prenatal appointments				0.629				< 0.05				< 0.05				0.348
1–3	320	63.0	508		67	47.9	140		347	60.9	570		23	57.5	40	
4–5	668	63.4	1,054		136	40.6	335		964	70.5	1,367		81	58.3	139	
6 or more	2,790	64.6	4,319		1,647	50.8	3,240		3,688	80.0	4,608		807	63.7	1,267	
Total	3,778	64.2	5,881		1,850	49.8	3,715		4,999	76.4	6,546		908	63.0	1,446	
Number of previous pregnancies				< 0.05				< 0.05				< 0.05				< 0.05
Zero	1,632	65.4	2,495		939	55.2	1,701		2,130	77.0	2,765		458	67.9	675	
1	1,059	64.6	1,638		525	50.0	1,049		1,376	77.9	1,767		237	60.8	390	
2	553	63.6	869		244	44.8	545		817	77.4	1,056		126	57.3	220	
3 or more	536	61.0	878		142	33.8	419		676	70.6	957		91	55.8	163	
Total	3,780	64.3	5,880		1,850	49.8	3,714		4,999	76.4	6,545		912	63.0	1,448	

N: total of mothers per category for accuracy (between 2 kg/2 cm); n: total of mothers per variable; BMI: body mass index; BE: basic education; HS: high school; HE: higher education; PN: prenatal

* Defined as reported information between ± 2 units (kg or cm) for weight and height, between ± 1.4 units (kg/m^2^) for BMI, of the measured variable.

Regarding height, Table 3 shows greater accuracy among women with prenatal (PN) in the private sector, from the South region, white, and belonging to the A+B class.

**Table 3:** Distribution of mothers by variables selected in quartiles for sensitivity, according to height, pre-gestational weight, weight at the last appointment, and pre-gestational BMI. Brazil, 2011–2012.

Variable	Height					Pre-gestational weight					Weight at the last appointment					Pre-gestational BMI				
			
	N	Sensitivity (%)				N	Sensitivity (%)				n	Sensitivity (%)				n	Sensitivity (%)			
			
		1^st^ Q	2^nd^ Q	3^rd^ Q	4^th^ Q		1^st^ Q	2^nd^ Q	3^rd^ Q	4^th^ Q		1^st^ Q	2^nd^ Q	3^rd^ Q	4^th^ Q		Low weight	Adequate	Overweight	Obesity
Place of the PN	5,863					3,710					6,538					1,447				
Public service	5,302	85.9	56.6	61.6	82.0	2,774	85.5	67.7	66.5	87.1	5,175	93.8	76.2	94.2	92.9	1,280	44.8	81.1	74.1	90.6
Private service	314	90.2	58.1	71.2	94.2	787	86.1	76.8	83.4	87.2	1,138	91.4	73.1	94.2	97.2	95	50.0	79.0	75.0	53.3
Both	247	80.0	47.8	59.3	88.1	149	92.3	65.5	75.7	87.7	225	94.6	76.0	97.4	91.7	72	33.3	86.0	64.3	58.3
Geographic region	5,883					3,715					6,545					1,450				
North	793	83.6	73.7	62.2	48.2	266	91.4	67.9	69.3	83.3	529	91.7	89.4	70.2	97.0	167	72.7	94.4	46.8	68.4
Northeast	1,901	82.7	68.7	63.6	52.3	1,019	84.4	66.3	67.6	88.6	2,000	93.3	86.5	84.5	95.3	470	63.2	86.4	60.2	58.0
Southeast	2,003	75.6	68.3	69.5	63.9	1,616	80.0	57.0	79.7	91.1	2,658	91.6	92.6	80.5	97.3	470	92.0	86.9	55.1	84.2
South	811	86.9	77.4	70.2	66.8	677	86.6	71.4	79.0	93.3	1,107	88.4	89.4	83.2	97.9	263	87.5	88.4	55.3	68.8
Midwest	375	77.9	66.2	68.9	50.5	137	80.0	69.7	77.8	82.1	251	78.7	90.7	85.1	96.8	80	80.0	84.8	64.7	28.6
Age group (years)	5,877					3,711					6,540					1,447				
12–19	1,310	85.1	68.0	68.4	57.2	599	86.5	67.5	78.5	82.7	1,434	90.5	86.6	77.8	95.9	278	84.6	78.3	39.3	42.9
20–34	4,133	80.2	72.1	66.2	59.1	2,750	82.7	62.0	75.3	91.1	4,514	91.8	90.2	82.5	96.9	1,050	75.0	90.5	57.0	70.8
> 34	434	81.8	63.1	69.0	59.3	362	78.3	66.7	75.9	90.3	592	93.1	95.1	81.0	98.5	119	100	90.0	73.9	86.4
Race	5,887					3,790					6,489					1,448				
White	1,693	82.4	72.6	67.6	63.6	1,377	87.1	61.3	79.7	90.7	2,207	89.7	90.3	82.2	97.9	466	94.1	92.2	69.4	75.0
Black	494	62.9	67.9	72.4	58.9	260	79.1	67.6	69.6	94.0	555	91.7	89.6	81.9	98.2	105	100	87.5	31.1	87.5
Brown	3,601	82.7	68.9	65.5	56.8	2,030	81.8	64.3	74.0	90.0	3,693	92.0	89.2	81.2	96.1	861	72.0	85.1	55.1	67.6
Yellow	70	100	85.0	72.7	60.0	32	87.5	80.0	75.0	85.7	11	94.7	94.1	77.8	90.9	14	100	100	25.0	50.0
Indigenous	29	80.0	100	57.1	50.0	10	100	0	100	100	23	100	100	100	100	2	0	0	0	0
Marital status of the woman	5,882					3,715					2,519					1,447				
Without partner	1,126	82.6	62.3	65.8	57.7	546	73.7	47.0	65.9	94.7	1,228	92.0	91.7	80.4	98.7	181	81.8	88.9	53.7	85.7
With partner	4,756	81.0	72.2	67.2	59.7	3,169	85.4	67.0	77.1	90.1	1,291	91.2	89.1	81.9	96.7	1,266	78.7	87.6	56.7	69.8
Education level	5,860					3,697					5,061					1,444				
Incomplete BE	1,604	79.3	70.2	66.0	56.8	794	84.3	63.5	67.5	82.3	1,872	92.6	85.4	80.5	94.8	349	63.2	84.1	47.6	69.0
Complete BE	1,858	83.7	66.4	67.0	53.4	954	83.8	64.6	76.9	88.8	359	91.3	88.5	80.8	98.6	436	88.9	84.7	52.7	64.6
Complete HS	2,178	81.0	72.9	66.6	63.9	1,650	81.7	60.3	77.8	94.2	2,432	90.2	93.8	82.2	97.4	591	80.0	91.3	60.8	79.2
Complete HE or more	220	93.3	79.4	71.9	65.7	299	90.9	75.0	80.0	92.0	398	92.5	91.7	87.2	96.4	68	100	97.2	72.7	55.6
Economic class	5,830					3,692					6,497					1,438				
Classes A+B	837	80.5	70.3	69.3	66.3	910	86.2	73.8	82.3	93.7	1,260	89.9	90.5	82.4	98.0	234	90.9	86.7	65.0	64.3
Class C	3,322	80.9	70.6	67.5	60.1	1,997	82.5	62.0	73.9	90.8	3,532	90.0	91.0	81.8	96.5	862	82.9	88.6	56.2	73.9
Classes D+E	1,671	81.9	69.9	63.6	50.9	785	83.5	57.6	67.8	84.1	1,705	93.3	86.9	80.7	96.7	342	69.2	85.9	50.6	67.6
Number of PN appointments	5,879					3,714					6,545					1,445				
1–3	508	79.5	71.7	59.5	55.7	140	82.1	19.7	73.3	90.5	569	91.3	85.8	64.8	91.2	41	100	88.9	26.7	71.4
4–5	1,053	84.1	71.8	67.1	50.9	334	84.0	67.5	70.3	68.3	1,368	88.2	85.5	82.2	94.9	137	50.0	86.1	47.6	50.0
6 or more	4,318	80.9	69.8	67.7	61.5	3,240	83.5	66.3	76.4	92.2	4,608	92.9	91.6	82.9	97.8	1,267	83.6	87.9	58.8	72.6
Number of previous pregnancies	5,877					3,713					6,544					1,446				
Zero	2,495	78.8	70.4	59.1	61.4	1,702	81.7	63.5	79.8	94.2	2,765	91.6	89.6	81.1	97.2	673	88.6	89.8	47.1	67.9
1	1,638	81.2	70.5	66.8	60.8	1,049	87.4	63.1	76.2	89.1	1,766	89.9	91.2	83.0	97.5	390	57.9	83.6	64.4	71.1
2	868	84.0	73.2	59.4	50.0	544	77.6	67.5	70.7	89.9	1,057	92.8	92.6	82.9	95.5	218	71.4	90.3	63.2	80.0
3 or more	876	85.0	67.2	69.7	58.8	418	88.1	58.8	65.5	85.8	956	92.0	83.9	79.0	97.7	165	100	85.3	59.0	65.7
Total	5,884	81.4	70.3	66.9	59.5	3,715	83.5	63.4	75.7	90.5	6,543	91.4	89.6	81.7	97.1	1,448	79.2	87.7	56.2	71.6

BMI: body mass index; Q: Quartile; n: total of mothers per variable; n: total of mothers per category; BE: basic education; HS: high school; HE: higher education; PN: prenatal

For the pre-gestational weight of those who had prenatal care in the private sector, accuracy was higher in the group of Southeast residents, white, with higher education, and ≥ 6 prenatal appointments. For the weight at the last prenatal appointment, better results were found for women from the South or Southeast regions, with higher education, ≥ 6 prenatal appointments, adult, and up to two pregnancies. Regarding pre-gestational BMI, the mothers who were white, had higher education, and primigravida presented statistically significant differences in accuracy.

**Figure 2:**
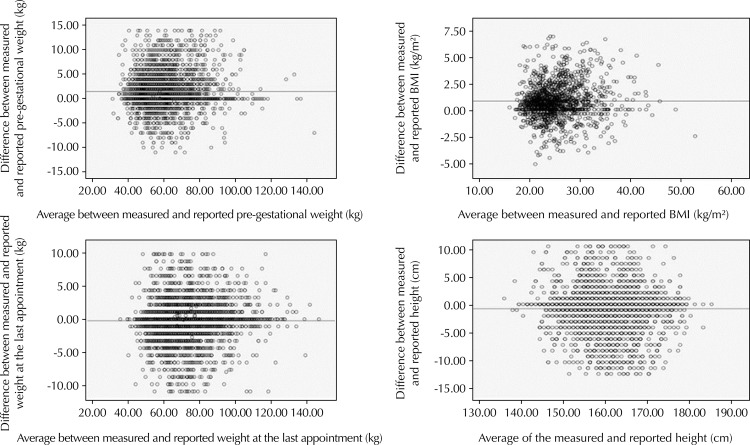
Differences between measured and reported anthropometric variables (pre-gestational weight, pre-gestational BMI, weight at the last visit, and height), according to the averages of the anthropometric variables in mothers. Brazil, 2011–2012.

In Figure 2, the Bland-Altman plot was used to show the difference between the measured and reported pre-gestational weight. The average difference of pre-gestational weight and the highest concentration of points are above the zero point, which shows an underestimation of the reported pre-gestational weight values, that is, women tend to report a lower pre-gestational weight. The same pattern can be observed for pre-gestational BMI. The Wilcoxon test was used to compare these measures, confirming the underestimation of the information.

Conversely, weight at the last appointment and height, according to the chart, show that the reported measures are overestimated by the mothers, that is, women tended to report greater weight at the last appointment and greater height than the measure of reference, which was present on the prenatal card. However, the Wilcoxon test showed that the differences between the measured and reported values were significant, even though most of the women reported the same value as the measured variable, both for weight at the last appointment and height.

The ICC showed high agreement between the measured and reported information for height (ICC = 0.898, 95%CI 0.880–0.912), pre-gestational weight (ICC = 0.957, 95%CI 0.930–0.971), weight at the last appointment (ICC = 0.988, 95%CI 0.987–0.988), and pre-gestational BMI (ICC = 0.922, 95%CI 0.871–0.948) (data not shown in tables).

Table 3 compares the reported and measured variables, divided into quartiles, for sensitivity analysis. For height, sensitivity was high in the first quartile. As the quartiles increased, the validity of information decreased, reaching 59.5% in the fourth quartile. For pre-gestational weight, we found the highest sensitivity in the fourth quartile.

Sensitivity for height indicated that the lowest percentages were among women who had prenatal appointments in both public and private services, in the North region, adolescents, brown, with complete basic education, and in class D+E. For pre-gestational weight, sensitivity was higher among women from private establishments, in the South region, and aged between 20 and 34 years.

For weight at the last prenatal appointment, sensitivity was generally high. In the first quartile, we found 91.5%, reaching 97.1% in the fourth quartile. When we evaluated the sensitivity of the strata, the lowest values were found among women from the North and adolescents.

Reported BMI showed a sensitivity of 88% for women with adequate classification and the lowest percentage was for overweight women; that is, the validity of the information was lower among overweight women, and, when we evaluated the sensitivity of the information using the variables selected by strata, we observed the lowest percentages of reported BMI.

## DISCUSSION

This study showed that most mothers accurately report their anthropometric data. The tendency to underestimate pre-gestational weight, as well as BMI, corroborates with the results of the literature^14^ ^,^ ^18^ ^,^ ^19^ ^,^ ^22^.

Weight at the last prenatal appointment was overestimated, but with a lower variation than that found for pre-gestational weight, which differs from the results found by Oliveira et al.^18^, in which pregnant women tended to underestimate the information. The lower variation found for weight at the last prenatal appointment may be related to memory, because of the lower interval between the last appointment (when weight was measured) and the information collected in the research. Considering that the interval between prenatal appointments decreases in the months before birth, women have greater access to prenatal care and information, which can improve their report^16^.

Women with lower weight and height tended to overestimate information, while those with greater weight and height tended to underestimate. The patterns established in search of a social ideal, generating a distortion of the self-image, can lead to errors when the information is reported, be it for weight or height^4^ ^,^ ^5^ ^,^ ^7^ ^,^ ^21^.

The overestimation of height, found in this study, has also been identified by other authors^4^ ^,^ ^8^ ^,^ ^10^ ^,^ ^18^. The shortest and highest women presented greater variation of information, differing from the results of Fonseca et al.^9^, who have found that the higher the individual, the smaller the difference found for this measure.

The accuracy of the information on reported height may change because of the presence of age-related bias. Younger women are measured only once in the gestation period by health professionals, who do not mind the fact that they are growing. Older women refer to a stature that they had in the past. Socioeconomic status and race may also contribute with the decrease in both the accuracy for height and weight, as they are associated with access to care and information about the health status. Therefore, non-white persons in less favorable conditions are those who have less accurate information^3^ ^,^ ^8^ ^,^ ^17^ ^,^ ^20^.

In the graphical analysis for pre-gestational weight and pre-gestational BMI, we observed a spacing between the points for women weighting approximately 70 kg and in the overweight range, respectively, in addition to a tendency for the underestimation of both measures, also observed in other studies^18^ ^,^ ^23^.

The ICC, which take systematic errors into account, were high for all anthropometric variables, showing almost perfect agreement and agreeing with other studies^9^ ^,^ ^14^.

Sensitivity values were high. Sensitivity showed a greater concordance of information for pre-gestational weight and weight at the last appointment, in the first and fourth quartiles, and for women who were classified as low weight and obese according to pre-gestational BMI, in agreement with the results of other studies^7^ ^,^ ^18^ ^,^ ^22^. This could be because women with inadequate weight (low or higher than expected) or inadequate pre-gestational BMI are diagnosed as with nutritional risk and are better monitored in the prenatal care; therefore, they present greater access to information and better perception regarding their anthropometric data.

In relation to height, the shortest women had better sensitivity and the tallest ones (fourth quartile) had a lower percentage of sensitivity, differing from the study of Boström and Diderichsen^4^, in which the lowest value was in the second quartile.

In this study, women who had prenatal care in the private service, more years of education, white or brown, from the South or Southeast regions, better economic classification, six or more appointments, and less parity presented the best results for validation of the anthropometric variables, reinforcing the strong relation between socioeconomic conditions and the quality of information^4^ ^,^ ^6^.

This validation study did not intend to be representative of the Brazilian population. However, the sample size allowed us to evaluate the validity of the information and possible differences between measured and reported measures^18^.

We highlight that, although the gold standard method used was the prenatal card, the differences between the information resembled those found in national and international studies that have obtained the measures directly, showing that the card is a relevant instrument for the anthropometric evaluation of pregnant women.

The lack of records of the anthropometric variables on the card limited the inclusion of more women who could represent the Brazilian population. However, as the anthropometric data presented high agreement for the self-reported measures, they could be used to outline the nutritional profile of women in the gestational period, as well as their weight gain, allowing their use in population-based studies when no resources for measurement are present.
